# A First Step to a Biomarker of Curative Surgery in Colorectal Cancer by Liquid Biopsy of Methylated Septin 9 Gene

**DOI:** 10.1155/2020/9761406

**Published:** 2020-06-05

**Authors:** M. Leon Arellano, M. García-Arranz, R. Ruiz, R. Olivera, S. Magallares, S. Olmedillas-Lopez, T. Valdes-Sanchez, H. Guadalajara, D. García-Olmo

**Affiliations:** ^1^Department of Surgery, Hospital Fundación Jimenez Diaz, Madrid, Spain; ^2^New Therapy Laboratory, Instituto de Investigación Sanitaria Fundación Jiménez Díaz, Madrid, Spain; ^3^Bemygene, Valencia, Spain

## Abstract

**Objectives:**

To confirm that patients affected by colorectal cancer have the V2 region of Septin 9 (*SEPT9*) gene hypermethylated in the circulating free DNA from a peripheral blood sample before surgery and to determine if this hypermethylated DNA disappears from the patients after complete resection of the tumour.

**Methods:**

Plasma from 10 patients with colorectal cancer was collected preoperative and three months after surgery. The analysis of the methylation status of the promoter region of the *SEPT9* gene was performed using a 7500 Fast Real-Time PCR System.

**Results:**

Hypermethylation of *SEPT9* gene was detected in 8 out of 10 preoperative samples (one negative result was probed to be a Lynch syndrome) and in 4 out of 10 postoperative samples matching with the cases of recurrence or persistence of disease. This means that, in this sample, the preoperative sensitivity and specificity of the test were 88.9% and 100%, respectively, and there is 100% correlation between the positive results of the *SEPT9* test and a recurrence/persistence of the disease in patients after surgical resection.

**Conclusions:**

Our study shows that circulating hypermethylated *SEPT9* is a specific colorectal cancer biomarker. This hypermethylated *SEPT9* DNA disappears around three months after surgery and that circulating hypermethylated *SEPT9* may be the first noninvasive marker for postsurgical diagnosis; this conclusion must be confirmed with a more significant number of patients.

## 1. Introduction

Colorectal cancer (CRC) is one of the most frequent tumours and almost the leading cause of deaths among cancer worldwide. Despite the development of new treatments, most patients are first-time diagnosed at the middle or late stage of CRC, leading to high mortality and a poor prognosis. The surgical approach is the best treatment for patients with CRC, but recurrence or persistence after resection is associated with a severe prognosis [1].

Approximately 25% to 40% of patients who undergo curative resection of colorectal cancer (CRC) develop tumour recurrence with eventual demise [2]. An optimal surveillance protocol for CRC includes CT scans, colonoscopies, and serum carcinoembryonic antigen (CEA) measurement, although this blood marker has a reduced sensitivity or specificity. Normal CEA values may be found in almost 50% of cancers before surgical resection and often do not rise during recurrences [3]. The primary objective of a surveillance protocol of CRC patients is to improve survival rates [4].

Therefore, this leads to a search for an accurate, convenient, and noninvasive biomarker that could detect persistence or recurrence of CRC, including also patients with a complete response in rectal cancer after neoadjuvant therapy, evaluation of complete resection after peritoneal oncological surgery, or local excision for early stage rectal cancer. With this tool, we could offer better surveillance and a rational adjuvant prescription.

The *SEPT9* gene methylation assay, a blood-based test explicitly used for CRC detection and screening, was developed and used clinically in the last decade [5, 6]. The test analyses the methylation status of the gamma promoter region of transcript V2 of the *SEPT9* gene. Because it is known that CRC develops from the accumulation of changes at the genetic and epigenetic levels in the epithelial cells of the colon, molecular markers of genetic and epigenetic alterations in tumour tissues and peripheral blood have been evaluated. The hypermethylated *SEPT9* gene has emerged as an accurate biomarker to detect CRC in peripheral blood and tumoural tissue [7, 8]. This test analyses the methylation status of the gamma promoter region of the *SEPT9* gene V2 transcript, which is differentially methylated in CRC patients. Septins are an evolutionarily conserved group of GTP-binding proteins that form filamentous hetero-oligomers, but their molecular role in tumorigenesis remains largely unknown [9–12]. Septin genes are reported to be essential in cell division, especially in cytokinesis, but they are also involved in membrane transport and fusion and exocytosis; they can also act as scaffolds that recruit other proteins or provide rigidity to cell membranes [13–15].

*SEPT9* promoter is hypermethylated in CRC tissues but also can be detected in blood plasma, due to the presence of tumour DNA released from necrotic and apoptotic CRC cells [6, 7, 16]. Numerous validation studies analyzing the performance of the *SEPT9* assay have been conducted; in particular, the FDA-approved test Epi proColon 2.0, developed by Epigenomics AG, reached sensitivity and specificity values ranging from 68% to 96% and from 80% to 97%, respectively [17]. For these reasons, it has emerged as an accurate and noninvasive method for primary CRC detection. The Septin 9 assay was initially developed for the early diagnosis of CRC in population screening, especially for those reluctant to undergo colonoscopy. Later on, several reports have also suggested its usefulness in monitoring patient evolution following a therapeutic intervention or even as predictive markers of the response to chemo- and radiotherapy [18, 19].

Although the test was designed for early detection and screening of CRC, due its sensitivity and the high specificity found, the hypothesis of using it as a diagnostic tool as a biomarker of detection of curative surgery and as a recurrence follow-up protocol seems reasonable. We decided to perform a proof of concept study measuring the *SEPT9* methylation level before and after surgery on ten patients with the following objectives:

1. To assure if indeed those patients affected by CRC, before surgery, have the *SEPT9* promoter hypermethylated in the circulating free DNA from a peripheral blood sample
2. To check if *SEPT9* hypermethylated DNA disappears after a complete resection of a tumour
3. To determine the relationship between the maintenance of hypermethylated DNA after a complete resection of the tumour and the possible recurrence or persistence of CRC

## 2. Material and Methods

### 2.1. Patients and Sample Collection

A prospective study of 10 patients with CRC at a single institution (Hospital Universitario Fundación Jimenez Diaz) was conducted between March 2017 and December 2017. The study was approved by the Ethics Committee Clinical Research (PIC number 157_2016_FJD), and all the patients included signed the informed consent prior the extraction of the samples.

Patients included in the study must meet the following inclusion criteria: a confirmed diagnosis of colorectal cancer, surgical treatment of a tumour without the need for a posterior ostomy, no previous treatment of chemotherapy or radiotherapy, age between 50 and 75 years, and signed informed consent.

Blood samples were collected at two points: preoperative and three months after surgery. All patients had the established protocol of postoperative CRC surveillance. It was recorded in the clinical history all the values associated with the characteristics and evolution of patients during the clinical follow-up (tumour stage, adverse events, clinical progression, and pain).

Ten mL of blood samples were extracted from each patient into potassium ethylenediaminetetraacetic acid (EDTA) tube and processed immediately (less 1 hour) by double centrifugation at 1400 × g for 12 min. The plasma obtained was transferred into a new tube and directly stored at -80°C for subsequent testing.

### 2.2. Analysis of the Methylation Status of Circulating *SEPT9* DNA in Plasma

The circulating free DNA (cfDNA) from 3.5 mL of plasma was captured, concentrated, and bisulfite-converted using the Epi proColon 2.0 Plasma Quick Kit following the instructions of the manufacturer (Epigenomics AG, Berlin, Germany). The ammonium bisulfite (Epi proColon Bisulfite Solution) converts unmethylated cytosines into uracils. The bisulfite-converted DNA (bisDNA) was then amplified in a duplex quantitative PCR (qPCR) using the Epi proColon Sensitive PCR kit. The kit includes specific primers for the promoter region of *SEPT9* and *ACTB* (*β*-actin), the latter as an internal control for DNA concentration, which hybridise to regions lacking CpG dinucleotides. The PCR reaction is based on a heavy methyl amplification, in which a bisulfite-converted unmethylated sequence-specific blocker prevents the amplification of unmethylated DNA, combined with the use of a methylated *SEPT9*-specific fluorescent detection probe [9]. The samples were amplified in triplicate using a 7500 Fast Real-Time PCR System (Thermo Fisher Scientific). Epi proColon positive and negative external controls were used in all independent runs.

We recorded PCR data from the 7500 Fast Dx software for *ACTB* and methylated *SEPT9* for each of the triplicate reactions and then analyzed the *SEPT9* and *ACTB* cycle threshold (Ct) within 45 cycles of amplification. Results were considered valid when the *ACTB* Ct was ≤32.1, and the external negative and positive controls met the validity criteria specified by the manufacturer. A *SEPT9* Ct <45 cycles were considered a positive result, while an undetermined Ct was taken as a negative result. Any other *SEPT9* Ct value was deemed to be invalid (Table 1).

### 2.3. Recurrence Criteria

Local recurrence or persistence was defined as a clinical, radiological, and pathological evidence of the same histological tumour type at the region of the anastomosis. Distant recurrence or persistence was defined as clinical or radiological evidence of systemic spread outside a primary tumour.

Two entirely independent teams were created, on one side, the surgical group, which selected the patients and had access to the clinical history, and on the other side, the laboratory group, which processed the blood samples obtained from patients without access to clinical information. Once all the tests were done, both groups met to analyze their knowledge and draw the conclusions of this proof of concept. To assure the blind process of sample collection and manipulation, the results were revealed after two samples of the 10 patients surveillance analysis were completed.

## 3. Results

Median age at diagnosis was 67.5 (range, 53–75 years), six males and four females. Regarding tumour localization, there are three at sigmoid colon, six at right colon, and one at the left colon. Tumour stage: 2 patients were T4N2M1, one patient was T4N0, one patient was T3N2M1, two patients were T3N1, three patients were T3N0, and one patient was T1N2.

After inclusion, all patients were treated by resection of the tumour.

A positive result in the *SEPT9* blood test was obtained in 8 of the 10 preoperative samples analyzed, indicating the presence of hypermethylated *SEPT9* DNA in plasma (Table 2). After a clinical and pathologic analysis, following the Amsterdam criteria for hereditary nonpolyposis colorectal cancer, one of the negative cases was probed to be a Lynch syndrome, which may explain the absence of hypermethylated *SEPT*9 DNA in the plasma sample from this patient.

The *SEPT9* blood test was positive in 4 of the 10 postoperative samples. These cases correlated with four recurrences/persistences of CRC, including a case of hepatic, peritoneal, lung, and lymph node metastases, which were confirmed by clinical, pathological, and radiological outcomes. The other six patients with a negative result were free of disease after a median follow-up period of 26 months (range, 25-29 months) (Table 3).

It should be noted that one case had a concomitant urologic neoplasm at the preoperative and postoperative test period. In this case, a negative result was obtained in the 3-month postoperative *SEPT9* test, supporting the specificity of this test for CRC detection.

In our sample, the preoperative sensitivity of the test was 88.9% and specificity 100%, while the 3-month postoperative sensitivity and specificity were 100%.

**Figure 1:**
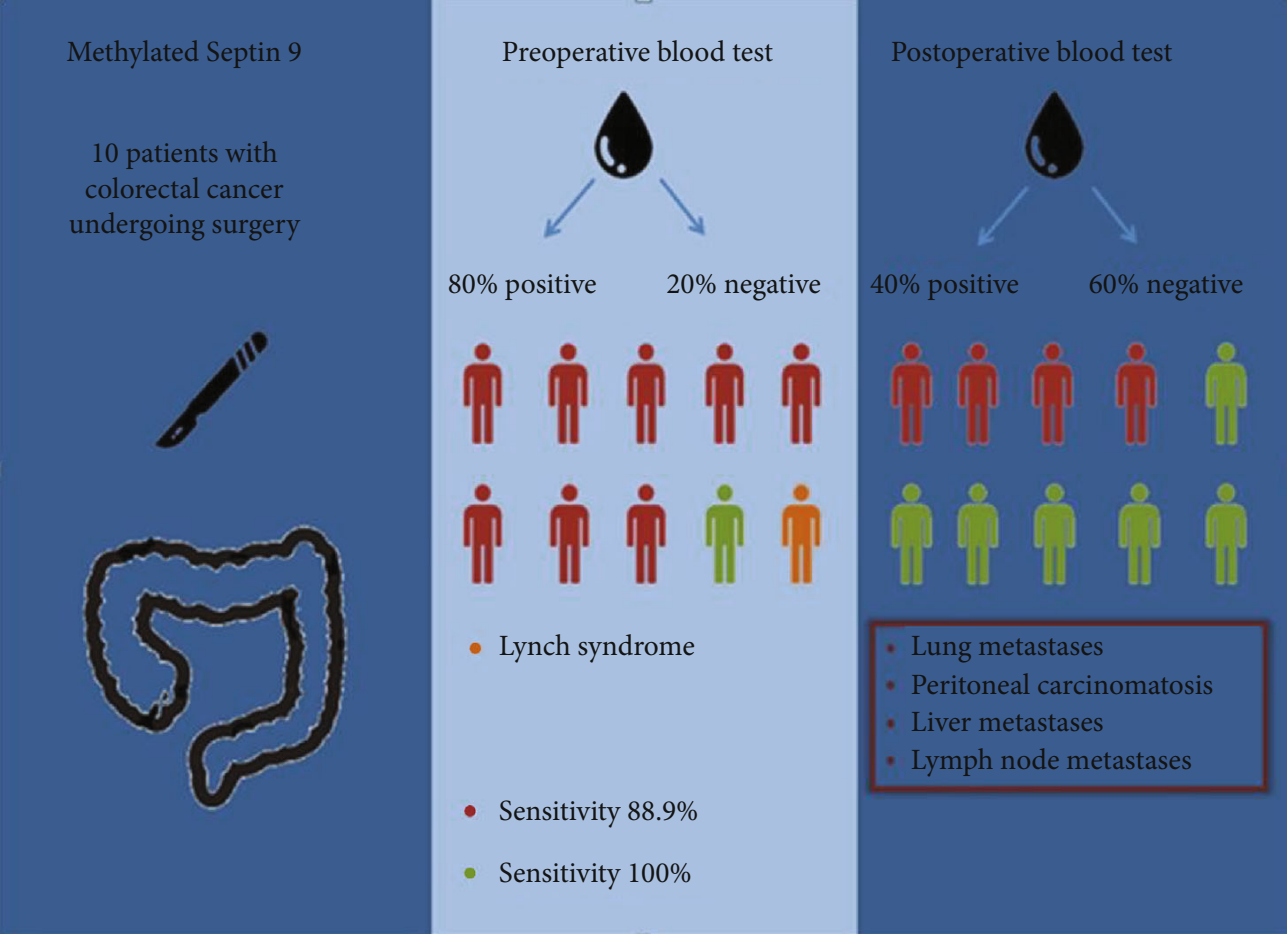
Sensitivity and specificity of a preoperative and postoperative blood test.

Preoperative and 3-month postoperative positive predictive values PPV were 100% (Figure 1).

## 4. Discussion

In this small sample, we obtained a preoperative methylated *SEPT9* blood test sensitivity and specificity values of 88.9% and 100%, respectively. The 3-month postoperative sensitivity and specificity were 100% given that the four positive cases in *SEPT9* blood test matched with the four recurrences/persistence observed (Tables 2 and 3). This promising result leads us to think that the analysis of the methylation status of the V2 transcript of the *SEPT9* promoter could be a useful tool in the follow-up protocol of CRC patients.

Other pathways of colorectal carcinogenesis, like the mismatch repair genes in the microsatellite instability pathway [20], may not be detected by this test. The patient with Lynch syndrome could explain one of the negative results obtained in the preoperative *SEPT9* blood test.

Moreover, the presence of hypermethylated *SEPT9* DNA in plasma may be a specific biomarker for CRC even in the presence of other tumour lineages. We found that one patient with a concomitant urologic neoplasm and without a CRC recurrence/persistence had a negative result in the 3-month postoperative *SEPT9* blood test. This situation leads us to think that the methylation degree of *SEPT9* in circulating DNA may not be increased by other tumour lineages.

We think this test may be useful in other challenging clinical settings and not only as a follow-up test or as a preoperative screening result. Our results indicated the hypermethylation of *SEPT9* could be useful as an epigenetic biomarker for total remission after neoadjuvant therapy in locally advanced rectal cancer, especially in a watch and wait management protocol. This tool can be added to the radiologic and clinical assessment after a complete response.

Also, it could be used as a measuring tool to confirm a complete resection after oncological surgery for peritoneal carcinomatosis from CRC or even after transanal microsurgery in early stage rectal cancer to assure the complete excision and as a noninvasive follow-up tool.

The proposed test seems to demonstrate a high percentage of success with the added advantage of being a minimally invasive procedure that obtained results in less than 96 hours.

We also consider it necessary to analyze the time limit after surgery in which we can detect the hypermethylation of *SEPT9* DNA in plasma without loss of prognostic value. In our study, we proposed three months to ensure a complete removal of the CRC circulating-free DNA with a hypermethylated *SEPT9* gene from peripheral blood vessel. Unfortunately, this time is too long to decide on the future treatment of patients. Therefore, new studies must be carried out to look for the time limit when the methylated gene is eliminated from the blood.

Our initial data is presented as the first noninvasive alternative to determine a complete or curative surgery in patients after undergoing colorectal cancer treatment. Further investigation, with a higher number of patients, is needed to validate the results we have obtained in this study.

## Figures and Tables

**Table 1:** Criteria for the validity of the system according to manufacturer instructions. Ct is threshold amplification cycle. *ACTB* is *β*-actin gene.

Gene	Results	Conclusion
*SEPT9*	Ct < 41.1	Control positive valid
*ACTB*	Ct ≤ 29.8	
*SEPT9*	Ct undetermined	Control negative valid
*ACTB*	Ct ≤ 7.2	

**Table 2:** Results and validity of preoperative and postoperative *SEPT9* blood test.

Patient	Preoperative			Postoperative		
	*SEPT9* (Ct)	*ACTB* (Ct)	Results	*SEPT9* (Ct)	*ACTB* (Ct)	Results
1	36.1 ± 0.1	29.5 ± 0.1	Positive	Undetermined	27.7 ± 0.0	Negative
2	32.2 ± 0.3	28.4 ± 0.2	Positive	40.6 ± 0.1	27.1 ± 0.1	Positive
3	40.3 ± 1.8	29.1 ± 0.1	Positive	Undetermined	28.3 ± 0.1	Negative
4	28 ± 0.1	29.0 ± 0.1	Positive	33.6 ± 0.5	26.5 ± 0.1	Positive
5	36.9 ± 0.4	30.8 ± 0.2	Positive	37.2 ± 0.8	27.9 ± 0.1	Positive
6	39.6 ± 0.3	28.3 ± 0.0	Positive	Undetermined	29.3 ± 0.2	Negative
7	37.2 ± 0.8	27.9 ± 0.1	Positive	40.4 ± 0.1	27.4 ± 0.1	Positive
8	Undetermined	29.3 ± 0.2	Negative	Undetermined	28.3 ± 0.1	Negative
9	Undetermined	29.8 ± 0.1	Negative	Undetermined	27.4 ± 0.0	Negative
10	37.0 ± 0.9	29.4 ± 0.3	Positive	Undetermined	27.9 ± 0.1	Negative

**Table 3:** Clinical, tumour stage and localization compared with the results of *SEPT9* blood test in preoperative and postoperative samples. M: male; F: female.

Patient	Age	Sex	Tumour stage	Localization	Preoperative *SEPT9*	Recurrence/persistence	Postoperative *SEPT9*
1	60	M	T3N1a	Right colon	Positive	No	Negative
2	74	F	T1N2a	Right colon	Positive	Retroperitoneal lymph nodes metastases	Positive
3	75	M	T3N1a	Left and urologic tumour	Positive	No	Negative
4	66	F	T4N2M1b	Sigmoid colon	Positive	Liver metastases	Positive
5	66	M	T3N2M1b	Right colon	Positive	Peritoneal carcinomatosis	Positive
6	73	F	T4N0	Sigmoid colon	Positive	No	Negative
7	73	F	T4N2M1b	Sigmoid colon	Positive	Pulmonary metastases	Positive
8	53	F	T3N0 Lynch syndrome	Right colon	Negative	No	Negative
9	63	F	T3N0	Right colon	Negative	No	Negative
10	72	F	T3N0	Right colon	Positive	No	Negative

## Data Availability

The data used to support the findings of this study are included within the article.
